# Comparison of Two Post-Stroke Rehabilitation Programs: A Follow-Up Study among Primary versus Specialized Health Care

**DOI:** 10.1371/journal.pone.0166242

**Published:** 2016-11-11

**Authors:** Remedios López-Liria, Francisco Antonio Vega-Ramírez, Patricia Rocamora-Pérez, José Manuel Aguilar-Parra, David Padilla-Góngora

**Affiliations:** 1 Department of Nursing, Physiotherapy and Medicine, University of Almería. Almería. Spain; 2 Complejo Hospitalario Torrecárdenas. Servicio Andaluz de Salud. Almería. Spain; 3 Department of Psychology, University of Almería. Almería. Spain; IRCCS E. Medea, ITALY

## Abstract

**Objective:**

To compare home-based rehabilitation (RITH) and standard outpatient rehabilitation in a hospital setting, in terms of improving the functional recovery and quality of life of stroke patients.

**Study Design and Setting:**

This was a prospective cohort study in Andalusia (Spain).

**Participants:**

One hundred and forty-five patients completed the outcome data.

**Measures:**

Daily activities were measured by the Barthel index, Canadian Neurological Scale (to assess mental state), Tinetti scale (balance and gait), and Short Form Health Survey-36 (SF-36 to compare the quality of life).

**Results:**

No statistically significant differences were found between the two groups regarding the clinical characteristics of patients in the initial measurement, except for age and mental state (younger and with greater neurological impairment in the hospital group). After physical therapy, both groups showed statistically significant improvements from baseline in each of the measures. These improvements were better in RITH patients than in the hospital patients on all functionality scales with a smaller number of sessions.

**Conclusions:**

Home rehabilitation is at least as effective as the outpatient rehabilitation programs in a hospital setting, in terms of recovery of functionality in post-stroke patients. Overall quality of life is severely impaired in both groups, as stroke is a very disabling disease that radically affects patients’ lives.

## Introduction

Stroke is a neurological disease that has a significant social and economic impact [1]. In Spain, stroke is the leading cause of death among women, the second leading cause of death in males, and the most frequent cause of permanent neurological sequelae in both sexes [2, 3]. Statistics confirm that one-third of those affected die within the first month, and it is estimated that the 44% of survivors are left with some functional disability [4,5]. Scientific literature shows that it is essential to track the progress of acute stroke rehabilitation and to understand the factors that may influence the prognosis of patients in the short and long term post-stroke [6,7].

Rehabilitation is a goal-oriented and time-limited process that allows patients to achieve an optimal functional level in physical, mental, and social fields, providing them with the tools needed to manage their own life [8,9]. Maintaining mobility in these patients is essential to prevent a decline in daily activities, re-hospitalizations, and unnecessary referrals to nursing homes. A suitable physiotherapeutic treatment that is adapted to a patient’s condition aims to achieve functional levels before stroke and to prevent deterioration, so that their health and quality of life can improve as much as possible [9,10]. To recover from a disease, it is essential to restore a sense of control and self-sufficiency in patients, so that patients feel that they can rebuild and reintegrate into society [11].

In recent years, there has been an organized or systematic emphasis on treatment of acute stroke patients that demonstrates its effectiveness in reducing dependency [12]. In the Andalusian Health Service, patients can receive physical therapy in the form of specialized care as outpatients (patients go to the hospital only to receive their rehabilitation treatment) or from primary health care in centers where there is a physiotherapist or a mobile rehabilitation and physical therapy team (RITH) [13]. In 2002, the Andalusian Regional Government integrated an RITH service into its Plan of Support for Andalusian Families, improving accessibility for patients and their families by offering it as an in-home service [14]. The reality of elderly people remaining at home and leading a life according to their abilities as they become less independent hinges on factors such as the degree of disability and functional dependence, socioeconomic status, the availability of housing that is accessible and safe, the support of family caregivers, and access to community and health services [15].

Investigations of home-based rehabilitation support in Spain have been made in the context of illnesses other than stroke [13, 15, 16], and they reflect that home care not only matches the quality of hospital care, but also offers patients the added benefit of the ease of being at home. Furthermore, the interventions are performed in a real-life scenario to which the patients can adapt according to their limitations [10, 15]. It is necessary to allow patients to participate in rebuilding their own lives by using strategies in rehabilitation that promote motivation and establish a therapeutic connection to their activities of daily life [9, 17].

Since stroke is such a significantly impactful disease, both nationally [2, 3, 10] and internationally [11, 18, 19], there is a need for further examination as to what are the most effective rehabilitation options for these patients. Moreover, there is a growing interest in cost-effective care [20], as health systems suffer increased economic pressures and prioritize a minimal length of in-hospital convalescence [21], even when the patients are still too dependent to live at home.

The main aim of this study was to compare RITH and standard outpatient hospital rehabilitation, in terms of improvement of the functional recovery and quality of life of patients who have suffered a stroke. We hypothesized that after discharge, home-based rehabilitation is at least as effective as hospital-based outpatient rehabilitation programs to correct/mitigate/ their initial level of disability.

## Material and Methods

### Design

This study was based on a prospective cohort study in the Clinical Management Unit of physical medicine and rehabilitation in Torrecárdenas Hospital, Almería province (Southern Spain) in the period between 2009 and 2012. This hospital, with its 732-bed capacity, provides both acute and post-acute care in any type of pathology.

### Subjects of Study/ Participants

Patients were consecutively recruited by the neurologist in the Stroke Unit at Torrecárdenas Hospital once they were referred for physiotherapy after being discharged. The definition of cerebrovascular disease (CVD) corresponds to the one established by the World Health Organization and the type of stroke was classified according to the criteria of the *Oxford Community Stroke Project* [8].

The number of subjects required for this study was calculated across a sample of patients who received RITH for stroke [16], from 2004 to June 2009, included in a previous study in patients with similar characteristics, using Epidata software version 3.1, for comparison of independent averages. A standard deviation was obtained from initial and final Barthel scores [22], 23.788 for the first group and 32.993 for the second group; the mean difference detected was 14.710; considering for calculation a ratio among samples of 1, a confidence level of 95%, and a power of 80%. The results showed a minimum sample size of 61 patients; considering a possible loss of 10%, the number of subjects to recruit would be 67 patients for each group (RITH and Hospital).

### Inclusion and Exclusion Criteria

Inclusion criteria were patients who were referred for physiotherapy using RITH or in Torrecárdenas Hospital after acute stroke, with premorbid ability to live at home. Generally, the requirements for referral to RITH have to include the existence of infrastructural barriers, multiple comorbidities that contribute to functional impairment or a state of medical instability [14]. On the other hand, patients can be referred to the Hospital-based program from neurology, rehabilitation services, or other specialists, when a rehabilitation process is considered necessary.

Exclusion criteria for both physical therapy programs were: the patient could travel by himself, or was not considered in a dependent state in terms of activities of daily living (ADL) (Barthel Index [22] score of 91 or better); no treatment acceptability by the patient or the family; lack of cooperation from the patient or caregivers; severe cognitive impairment leading to failure to understand and act upon instructions; inability to understand or speak the Spanish language; patients with terminal disease; a lack of need for physiotherapy; or an adjunctive physiotherapy treatment had been performed at a different institution.

**Fig 1:**
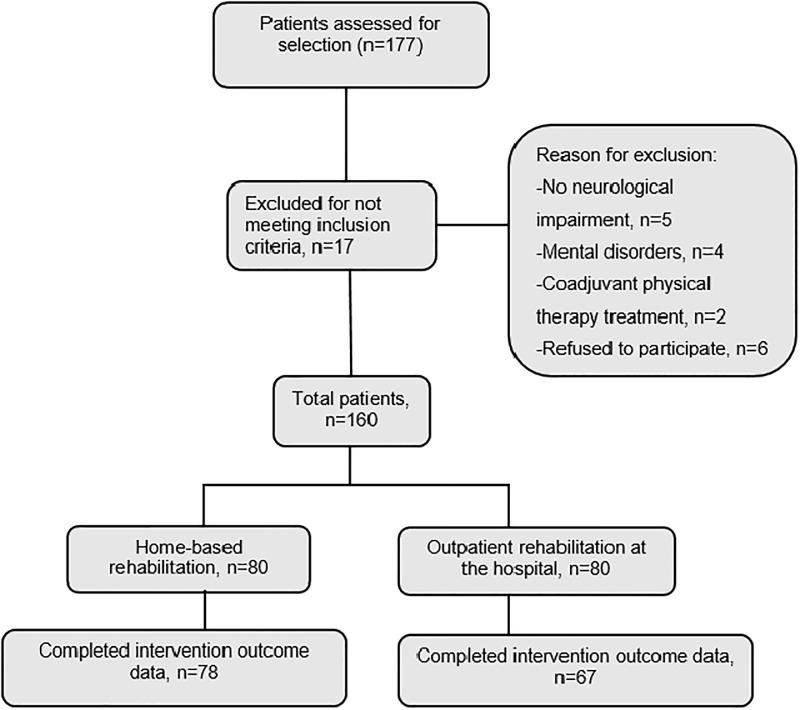
Flow Chart.

The treatment scenario (hospital or home) was selected by a rehabilitation physician, mainly on the basis of the need for assistance in ADL, the characteristics of patients’ homes (architectural barriers), and the availability (or lack) of social and family support, in accordance with the Rehabilitation Method Guidelines of Primary Care in Andalusia [14]. Eventually, the total sample was composed of 145 patients, 78 of which constituted the RITH group and 67 the hospital group (in the Department of Rehabilitation and Physical Therapy). Enrollment for the study is described in the flow chart (Fig 1). All patients provided written informed consent before treatment in accordance with the Helsinki Declaration. This study was approved by the Scientific Ethics Committee of the Torrecárdenas Hospital Complex and the Research Commission of the Almería Health District (PI-0449), and adhered to the guidelines of the International Committee of Medical Journal Editors.

### Outcome measures

Premorbid and baseline characteristics were recorded at the time of enrollment. To determine the impact of either home-based or outpatient physical therapy on functional independence achieved post-stroke, various validated scales were used: the Barthel Index to measure ADLs [22], the Canadian Neurological Scale to assess mental state [23], and the Tinetti Scale to assess balance and gait [24]. The SF-36 was used to compare the quality of life of the two groups of patients [25].

All subjects were assessed by an external physiotherapist who did not participate in the patients’ treatment within the first week after the stroke and after the end of training rehabilitation. Other secondary variables analyzed were the rehabilitative goals, the number of sessions, and the number of hospital readmissions.

### Rehabilitative Process

In this study, all patients received early physical therapy in their room, during their hospital stay. It has been shown that rehabilitation must start as soon as a diagnosis is established, a check of vital status has been carried out, and within the first week post-stroke to ensure the best results [12]. Therefore, bedridden patients are treated in the hospital from the start of the stabilization process by a physical therapist in the neurology unit. During this phase of bed rest, early mobilization reduces the complications that can arise from immobility. The active participation of the patient in movement therapy is encouraged to the extent possible.

In the motor area, the deficit caused by stroke is usually unilateral (hemiparesis or hemiplegia), and enabling techniques are used, such as the Bobath method [26] that counteracts spasticity by inhibiting postures obtained through slow mobilization in the opposite direction to the one imposed by spasticity, and in a proximo-distal progression.

The registration and information form developed in the unit contains the main treatment goals for the acute, sub-acute, and chronic phase, as classified by physiotherapists. Generally, normalization of postural tone and selective mobility training will be applied at first mainly on the trunk and lower limbs, in preparation for gait. Subsequently, the focus is on the integration of the upper limbs in ADL.

Patients in the RITH group were treated by a team that included a physiotherapist, an occupational therapist, and a rehabilitation doctor, each of whom had prior experience in stroke treatment (these professionals didn’t know about patients’ participation in the study). For each patient, home training was based upon both the individual, particular needs and the rehabilitation goals. Patients in the hospital group were treated according to the standard rehabilitation procedure in the Stroke Hospital Unit by physiotherapists (who didn’t know about the study) in individual training sessions that focused on the patient’s problems. This type of treatment typically ends when the rehabilitation doctor believes that there are no new functional objectives to reach, or when a patient no longer wants to continue.

### Data Analysis

The results were analyzed using Statistical Package for Social Science (SPSS) version 21.0, by technical staff who did not participate in the data collection or the study design, and who had no competing interests. Descriptive statistics summarize the socio-demographic and clinical characteristics of participants, and display them as percentages.

T-tests were used to calculate the changes in physical, mental and social health outcomes that occurred during the recovery phase (within-group comparisons of pre- and post-intervention scores, T-test for related samples), and between the two groups (between-group comparisons of change scores in all outcome measures, T-test for independent samples). Parametric statistics were used for the continuous variables extracted by functional tests. A p value of < 0.05 was considered statistically significant.

## Results

Our analysis of the initial measurement data showed no statistically significant differences between the two groups in relation to any of the variables, except age (Table 1).

**Table 1:** Characteristics of RITH and Hospital groups of the study.

VARIABLES		RITH Group (n = 78)	Hospital Group (n = 67)	T Student test or Chi-square	Effect Size
		Average ± SD,%	Average ± SD,%	Test; P Value	
**AGE**		74.12 ± 10.78	64.49 ± 11.83	T = -5.122P < 0.001*	*d* = .850
**GENDER**	**Female**	47.4%52.6%	35.8%64.2%	χ^2^ = 1.995P = 0.158	*V* = .158
	**Male**				
**Ischemic Haemorrhagic CVD**		83.1%16.9%	83.6%16.4%	χ^2^ = 0.006P = 0.940	*V* = .006
**OBESITY**		15.1%	12.1%	χ^2^ = 0.255P = 0.613	*V* = .043
**HYPERTENSION**		80.8%	65.7%	χ^2^ = 4.597P = 0.100	*V* = .181
**ATRIAL FIBRILLATION**		15.1%	19.4%	χ^2^ = 0.462P = 0.497	*V* = .057
**ALCOHOL CONSUMPTION**		11.3%	11.9%	χ^2^ = 9.69P = 0.949	*V* = .046
**TOBACCO**		18.1%	22.4%	χ^2^ = 0.453P = 0.797	*V* = .057
**DIABETES MELLITUS**		30.2%	26.9%	χ^2^ = 1.257P = 0.739	*V* = .095
**PRIOR MIGRAINE**		8.3%	7.5%	χ^2^ = 0.317P = 0.854	*V* = .048

* P value represents the difference between RITH and hospital groups by comparing independent samples t-Student or chi-square for categorical variables.

SD = standard deviation, percentage %, p <0.05.

In terms of frequency of treatment per week, 46.2% of patients in the RITH group were treated three times per week, while the remaining 53.8% were treated two times per week. In the Hospital group, 40.3% of patients were treated two times per week, and the remaining patients were treated 3 times per week. Thus, no statistically significant differences in treatment frequency were found between the RITH and in-hospital rehabilitation services (t = 1.253; p = 0.212).

The treatment goals focused on minimization of the side effects of the deficits, recovery of the patient’s previous functional capacity, sequelae prevention, and equipping patients with strategies to adapt to their condition, as well as to their immediate environment. We have summarized the functional goals established by the rehabilitation professionals in Table 2, according to frequency (percentage of times each professional pointed to it as a goal of their treatment in each group). There were no statistically significant differences between treatment groups, except in the integration of the upper limbs in ADL and psychological stimulation; both had higher percentages in the hospital group.

**Table 2:** Functional Objectives for each group and comparison between them.

FUNCTIONAL OBJECTIVES	RITH	*Z*	Hospital	*Z*	*X*^*2*^	*p*	*V*
**Prevention of Vicious Attitudes**	70 (92.1%)	-0.4	62 (93.9%)	0.4	0.18	.751	.036
**Progressive verticalization in bed**	61 (80.3%)	-1.5	59 (89.4%)	1.5	1.60	.205	.126
**Control and extension of trunk in sitting position**	66 (86.8%)	-2.2	64 (97%)	2.2	3.46	.063	.182
**Normalise postural tone**	67 (88.2%)	-2.0	64 (97%)	2.0	2.70	.100	.164
**Facilitation Techniques in affected hemibody**	70 (92.1%)	-1.3	64 (97%)	1.3	1.65	.285	.105
**Improve altered sensitivity and perception**	64 (84.2%)	-1.8	62 (93.9%)	1.8	2.44	.118	.153
**Facilitate transition to standing position and assisted transfers**	73 (96.1%)	0.2	63 (95.55)	-0.2	0.03	1.00	.015
**Facilitate balance in standing position**	72 (94.7%)	-1.2	65 (98.5%)	1.2	1.58	.372	.101
**Integration of the upper limbs in ADL**	63 (82.9%)	-2.7	64 (97%)	2.7	5.99	.014*	.228
**Decrease pain**	34 (44.7%)	-0.1	30 (45.5%)	0.1	0.00	1.00	.007
**Decrease joint stiffness**	56 (73.7%)	-0.3	50 (75.8%)	0.3	0.00	.928	.024
**Teaching the use of canes/crutches**	51 (67.1%)	0.6	41 (62.1%)	-0.6	0.19	.657	.052
**Gait training**	73 (96.1%)	1.3	60 (90.9%)	-1.3	1.58	.303	.105
**Stairs**	65 (85.5%)	1.5	50 (75.8%)	-1.5	1.60	.206	.124
**Family training about patient’s gait training**	72 (94.7%)	0.2	62 (93.9%)	-0.2	0.04	1.00	.017
**Psychological stimulation**	38 (50%)	-5.3	60 (90.9%)	5.3	25.76	P<0.001*	.441
**Information for patients and families on measures of cooperation with treatment and ergonomic measures at home**	75 (98.7%)	1.2	63 (95.5%)	-1.2	1.38	.338	.097

* P value represents the difference between RITH and hospital groups, p <0.05.

Overall, the results of the questionnaires used in this research show that at the beginning of the study, both groups of patients showed significant ADL dependence, neurological impairment at the levels of consciousness, orientation and language, as well as altered balance and gait (Table 3). Both groups showed statistically significant improvements from baseline in each of the measures post-intervention.

**Table 3:** T-Test for related samples. Pre-test versus post-test, RITH group and Hospital group.

VARIABLES	Related test samples				Related test samples			
	Pre-test versus post-test				Pre-test versus post-test			
	RITH group				Hospital group			
	1^st^ Assessment Average ± SD, %	2^nd^ Assessment Average ± SD, %	t / mc nemar	P Value / Cohen d	1^st^ Assessment Average ± SD, %	2^nd^ Assessment Average ± SD, %	t / mc nemar	P Value / Cohen d
**BARTHEL INDEX**	39.41 ± 21.40	76.58 ± 21.16	-17.90	P < .001 (d = -1.74)	36.29 ± 25.39	58.64 ± 31.89	-9.92	P < .001 (d = -.775)
**CANADIAN SCALE**								
**1. Consciousness Level**								
**ALERT**	96.1%	98.1%		P = .625 (d = .186)	73.1%	85.1%		P = .039 (d = .296)
**SLEEPY**	3.9%	1.4%			26.9%	14.9%		
**2. Orientation**								
**ORIENTED**	85.5%	95.9%		P = .021 (d = .348)	58.2%	85.1%		P < .001 (d = .622)
**DISORIENTED**	14.5%	4.1%			41.8%	14.9%		
**3. Language**								
**NORMAL**	85.5%	95.9%	2.78	P = .007 (d = .372)	58.2%	85.1%	3.81	P < .001 (d = .397)
**EXPRESSION DEFICIT**	14.5%	4.1%			41.8%	14.9%		
**COMPREHENSION DEFICIT**	77.6%	90.5%			75.2%	84.6%		
**GLOBAL TINETTI**	1.92 ±1.81	19.28 ±5.82	-26.25	P < .001(d = -4.02)	6.14 ±7.96	14.39 ±10.41	-8.24	P < .001(d = -.890)
**GAIT TINETTI**	1.08 ±1.36	8.57 ±2.48	-25.45	P < .001(d = -3.74)	2.39 ±3.47	6.06 ±4.61	-7.36	P < .001(d = -.899)
**BALANCE TINETTI**	0.85 ±0.89	10.73 ±3.46	-25.27	P < .001(d = -3.91)	3.75 ±4.74	8.33 ±6.05	-8.19	P < .001 (d = .397)

Inter-group analysis was used to compare improvement in the two groups and the results are shown in Table 4. Patients in the RITH group achieved better scores on the Barthel Index, Canadian Scale, and Tinetti Scale, and had greater improvements from baseline scores on all functionality scales than the hospital group. Hence, the results demonstrated that the at-home rehabilitation patients were less disabled than the patients in the Hospital group after intervention.

**Table 4:** Test for independent samples. Descriptive statistics and significance of treatment effects between groups.

VARIABLES	RITH	HOSPITAL	DIFFERENCE INTER GROUPS	
	Average (SD)	Average (SD)	T / U /X^2^	Sig. +Cohen *d /r/v*
**BARTHEL INDEX**	Pre-test	39.41 (21.40)	36.29 (25.39)	-0.79 P = .428 / d = .132
	Post-test	76.58 (21.16)	58.64 (31.89)	
	Effect	37.17	22.35	-3.88 P < .001/ d = .662
**Canadian scale**				
**CONSCIOUSNESS LEVEL**	Pre-test	0.04 (0.19) X = 64.32	0.27 (0.44) X = 80.71	1962.50 P < .001/ r = .321
	Post-test	0.01 (0.11) X = 66.45	0.15 (0.35) X = 76.02	
	Effect	-0.03	-0.12	2142.50 P = .003/ r = .251
**ORIENTATION**	Pre-test	0.14 (0.35) X = 62.85	0.42 (0.49) X = 82.38	1850.50 P < .001/ r = .304
	Post-test	0.04 (0.19) X = 67.36	0.15 (0.35) X = 75.02	
	Effect	-0.10	-0.27	2209.50 P = .026/ r = .187
**LANGUAGE**	Pre-test	0.26 (0.52) X = 62.85	0.75(0.89) X = 82.38	11.49 P = .001/ *v* = .200
	Post-test	0.09 (0.29) X = 65.15	0.42 (0.76) X = 77.46	
	Effect	-0.17	-0.33	7.47 P = .006/ *v* = .162
**Tinetti scale GAIT (12 points)**	Pre-test	1.08 (1.36)	2.39 (3.47)	2.84 P = .006/ d = -.497
	Post-test	8.58 (2.47)	6.06 (4.61)	
	Effect	7.50	3.67	-3.92 P < .001/ d = .681
**BALANCE (16 points)**	Pre-test	0.85 (0.89)	3.75 (4.74)	4.81 P < .001/ d = -.850
	Post-test	10.64 (3.53)	8.33 (6.05)	
	Effect	9.79	4.58	-2.70 P = .008/ d = .466
**GLOBAL BALANCE**	Pre-test	1.92 (1.81)	6.14 (7.96)	4.15 P < .001/ d = -.731
	Post-test	19.24 (5.88)	10.41 (1.30)	
	Effect	17.32	4.27	-3.30 P = .001/ d = 2.07

Statistically significant differences were found between both rehabilitation groups in terms of the number of physiotherapy sessions given. The RITH patients received an average of 20.92 sessions, in comparison to an average of 28.86 sessions in the hospital group (t = 4.121; p<0.001). Overall, stroke patients treated at home required fewer treatment sessions than those treated in the hospital.

The average number of visits to the emergency room for complications during the recovery was under 1% in both the RITH and Hospital groups and the results from the Student’s t-test did not show statistically significant differences (t = -1.623; p = 0.7107) in terms of readmissions to the hospital (t = 1.430; p = 0.156).

The analysis of perceived quality of life, measured by SF-36 in both groups, is presented in Table 5. Patients from both the RITH and hospital groups reported similarly as to physical ability, physical pain, general health, vitality, social functioning, emotional role, and mental health. Generally, the most compromised aspects of quality of life were physical ability, and both physical and emotional role.

**Table 5:** Dimensions of SF-36 Health Survey in RITH and Hospital groups.

SF-36 Dimensions		RITH Group (n = 78)	Hospital Group (n = 67)			
		$\bar{x}$ (SD)	$\bar{x}$ (SD)	T Student	p	Cohen d
**Physical ability**	Pre-test	8.65 (15.68)	4.62 (14.12)	1.61	.108	.270
	Post-test	22.50 (24.00)	21.26 (31.61)	0.26	.789	.044
**Physical role**	Pre-test	0.32 (2.83)	0.00 (0.00)	0.92	.356	.159
	Post-test	7.69 (22.18)	16.41 (32.72)	1.90	.059	.311
**Physical pain**	Pre-test	40.67 (23.78)	44.08 (29.25)	0.77	.044	.128
	Post-test	54.56 (33.54)	52.07 (34.69)	0.43	.066	.072
**General health**	Pre-test	23.95 (11.72)	26.49 (14.55)	1.16	.246	.192
	Post-test	42.21 (23.63)	44.83 (19.81)	0.71	.474	.120
**Vitality**	Pre-test	28.33 (9.89)	33.35 (22.00)	1.81	.071	.294
	Post-test	38.91 (17.88)	40.29 (23.69)	0.39	.690	.065
**Social functioning**	Pre-test	22.27 (16.81)	32.08 (29.21)	2.52	.012	.411
	Post-test	40.70 (27.21)	44.96 (33.99)	0.83	.403	.183
**Emotional role**	Pre-test	6.83 (21.72)	12.43 (32.22)	1.24	.216	.203
	Post-test	22.22 (38.23)	27.86 (44.79)	0.81	.414	.135
**Mental health**	Pre-test	42.66 (12.87)	46.14 (21.77)	1.19	.235	.194
	Post-test	52.60 (18.61)	54.26 (20.11)	0.70	.438	.085
**PCS-36**	Pre-test	26.76 (5.07)	26.15 (5.35)	0.71	.482	.117
	Post-test	31.95 (8.01)	32.17 (8.18)	0.16	.870	.024
**MCS-36**	Pre-test	29.67 (7.48)	33.41 (13.22)	2.13	.034	.348
	Post-test	35.44 (10.70)	37.30 (13.17)	0.93	.349	.155

## Discussion

Despite a lower average of number of rehabilitation sessions, patients receiving treatment at home had better recovery and attained the same level of quality of life as the patients receiving hospital rehabilitation. In addition, RITH patients achieved higher scores on functionality scales than patients in the control group, although it is important to note that differences between the two groups were established in the initial assessments. For example, the patients in RITH group, despite being older, with an average age difference of 10 years, showed lower overall neurological impairment after stroke, assessed with the Canadian Neurological Scale (consciousness level, orientation, language), which could be a factor in their greater, more rapid recovery than those in hospital group.

Some epidemiological studies with patients who had suffered stroke [27, 28] considered similar variables as this study in order to determine the factors that are associated with the worst prognosis: socio-demographic factors, cardiovascular risk factors (tobacco or alcohol consumption, dyslipidemia, heart disease, diabetes, or a previous stroke), diagnosis, Barthel Index scores considering the level of autonomy and functional status of patients both premorbid and at discharge, and extent of neurological deficit using the Canadian Neurological Scale, both on admission and at discharge. A different study [29] has focused on developing an index to predict the likelihood of acute stroke patients being discharged home after hospitalization, by identifying eight independent predictors: being married, place of residence before hospitalization, physical independence, cognitive independence, the absence of liver disease, use of mechanical ventilation, non-oral feeding, and history of admission to intensive care units [29].

Recent studies [6, 9, 30] have reached the conclusion that early at-home rehabilitation programs after a stroke provided significantly better results in terms of physical function, decreased disability, increased quality of life, and reduced depression, in comparison with other typical care programs. One clinical trial [31] compared the changes in perceived health after 5 years of disease between patients who received rehabilitation at home and those who received conventional rehabilitation. They concluded that the long-term outcome is more favorable after rehabilitation at home. Data suggest that the environment is a key component to consider when evaluating the process of post-stroke rehabilitation [32, 33].

Our research demonstrates a positive outcome in patients of both groups in all evaluated areas: functionality, neurological scale, balance, and gait. Therefore, both services seem to be effective forms of post-stroke physical therapy. However, there were also some significant differences noted: before treatment application, we found younger and more affected patients in the hospital group, greater impairment in neurological area (measured with Canadian Scale), although regarding gait and balance the RITH group got worse scores in the initial assessment. It should not be overlooked that in most public health services in Spain there are criteria and protocols for patient referral to rehabilitation service in both Primary and Specialized Care, based on several variables including impairment, independence, or patient safety. This fact affects randomization of studies, making ethically difficult to design clinical trials on a disease such as CVD.

However, a systematic review [19] of post-stroke rehabilitation at home versus in other facilities such as a hospital, health center, or day center, found eleven studies where a significant positive effect in favor of rehabilitation at home was observed, at 6 weeks (P = 0.03) and at 3–6 months (P = 0.01). Some of these studies also reported better results for rehabilitation at home in terms of the overall cost and caregiver satisfaction. Another study found significant improvement in functional disability among stroke survivors during the recovery phase. The option of home-based rehabilitation should be available for those who are unable to move out of home or who will benefit from staying close to their community [34].

Gustafsson [35] performed a qualitative study to examine the experience of patients who had suffered a stroke and their caregivers, in the transitional time between hospital discharge and the first month back at home. The findings concluded that although patients and caregivers attested to the positive aspects of rehabilitation, they also expressed concern as to their limited ability to perform daily tasks safely, and admitted that customized strategies should be developed for the patient in preparation for discharge. Daily routines at home become troublesome because patients need more time to complete ADL, and caregivers must reconcile their family responsibilities with their own lives and priorities. In general, the results suggest that there is a need for stroke rehabilitation services to better support both patients and caregivers in this transition to home [36].

A recent study [36] aimed to determine whether the functional status or quality of life of stroke patients were good predictors to continue living at home, 2 years after the discharge from inpatient rehabilitation versus "institutionalization" or "death". Their results showed that 75% of survivors were still living at home 30 months after discharge.

The research presented here has some limitations, such as no adoption of criteria for blinding evaluators, or the fact that, prior to the treatment, there were differences between the compared samples, such as age, their initial impairment, or the physiotherapy services that provided treatment to the RITH and Hospital groups; these differences are inherent to the differentiation between Primary and Specialty Care. However, this study aimed to present a representative sample of current clinical practice in these services, so that there is no manipulation in the allocation of patients for their treatment or in the techniques used. In order not to fall into an overestimation of the results, it has to be taken into account that potential confounders like age, co-morbidity, home/community support were not controlled. However, the systematization of the data regarding the objectives of stroke treatment, can establish a basis for the design of new research to deepen the knowledge regarding stroke and can help to reduce the variability error, which is often noted in studies comparing the effectiveness of different treatments administered in the various locations where patients can be referred to. Descriptive studies as the one presented here can contribute data regarding important implications for clinical practice of health professionals.

It continues growing the evidence suggests that home rehabilitation is at least as good as outpatient rehabilitation programs in hospital, in terms of functionality achievement in patients. For future research it would be very interesting to include a cost-effectiveness analysis of both rehabilitation programs, in order to measure their effectiveness within the public health system. Determining the most appropriate context for the patient’s treatment, as well as understanding the impact of such context on the results, are two key priorities owing to the diversity of contexts for these therapies and the importance of optimizing the treatment and diversity of resources available for this purpose. Rehabilitation in the home services should cover evenly across the Spanish geography, attending both social and health needs by providing Physiotherapy to those patients with major functional limitations and comorbid conditions, or where the transfer and transportation to care services is difficult. Rehabilitation in the home has been found to help to reduce the impact of the disease, avoiding exacerbation of diseases, consequences of immobility and caregivers' overload. However, the provision of this service is not the same throughout the Spanish territory, due to the different health policies of each community and problems of geographical dispersion. So it is still necessary to investigate the difficult situation of those patients who, under similar circumstances of demand and extreme need of care and palliative care, have no access to this service.

## Conclusions

This study observed a significant improvement in patients attending both physiotherapy services, which was measured by different scales that measured functionality, consciousness, orientation, language, balance, and gait. All patients were discharged from the service after improvement, with a considerable increase in their functional independence scores. The data showed that RITH group patients had better results with respect to recovery and had fewer sessions when compared with the Hospital group patients. Quality of life was severely impaired similarly in both groups, as stroke is a very disabling disease that radically changes the patients’ life, making them highly physically dependent; however, the degree of dependence can be minimized with an early, proper rehabilitation program.

## Supporting Information

S1 Dataset Strokenglish. (SAV)
